# Clinical evaluation of [^18^F] JNJ-64326067, a novel candidate PET tracer for the detection of tau pathology in Alzheimer’s disease

**DOI:** 10.1007/s00259-020-04880-1

**Published:** 2020-06-13

**Authors:** Mark E. Schmidt, Luc Janssens, Diederik Moechars, Frederik J. R. Rombouts, Maarten Timmers, Olivier Barret, Cristian C. Constantinescu, Jennifer Madonia, David S. Russell, Christine M. Sandiego, Hartmuth Kolb

**Affiliations:** 1 Janssen Research & Development, Janssen Pharmaceutica NV, Beerse, Belgium; 2 Invicro, a Konica Minolta company, New Haven, CT USA; 3 Present Address: Laboratory of Neurodegenerative Diseases, Molecular Imaging Research Center, French Atomic Energy Commission, Fontenay-aux-roses, France; 4 Present Address: Biohaven Pharmaceuticals, New Haven, Connecticut USA; 5 Janssen Research & Development, La Jolla, CA USA

**Keywords:** Alzheimer’s disease, Tau aggregates, PET, Isoquinoline

## Abstract

**Purpose:**

The accumulation of misfolded tau is a common feature of several neurodegenerative disorders, with Alzheimer’s disease (AD) being the most common. Earlier we identified JNJ-64326067, a novel isoquinoline derivative with high affinity and selectivity for tau aggregates from human AD brain. We report the dosimetry of [^18^F] JNJ-64326067 and results of a proof-of-concept study comparing subjects with probable Alzheimer’s disease to age-matched healthy controls.

**Methods:**

[^18^F] JNJ-64326067 PET scans were acquired for 90 min and then from 120 to 180 min in 5 participants with [^18^F]-florbetapir PET amyloid positive probable AD (73 ± 9 years) and 5 [^18^F]-florbetapir PET amyloid negative healthy controls (71 ± 7 years). Whole-body [^18^F] JNJ-64326067 PET CT scans were acquired in six healthy subjects for 5.5 h in 3 scanning sessions. Brain PET scans were visually reviewed. Regional quantification included kinetic analysis of distribution volume ration (DVR) estimated by Logan graphical analysis over the entire scan and static analysis of SUVr in late frames. Both methods used ventral cerebellar cortex as a reference region.

**Results:**

One of the healthy controls had focal areas of PET signal in occipital and parietal cortex underlying the site of a gunshot injury as an adolescent; the other four healthy subjects had no tau brain signal. Four of the 5 AD participants had visually apparent retention of [^18^F] JNJ-64326067 in relevant cortical regions. One of the AD subjects was visually negative. Cortical signal in visually positive subjects approached steady state by 120 min. Temporal and frontal cortical SUVr/DVR values in visually positive AD subjects ranged from 1.21 to 3.09/1.2 to 2.18 and from 0.92 to 1.28/0.91 to 1.16 in healthy controls. Whole-body effective dose was estimated to be 0.0257 mSv/MBq for females and 0.0254 mSv/MBq for males.

**Conclusions:**

[^18^F] JNJ-64326067 could be useful for detection and quantitation of tau aggregates.

**Electronic supplementary material:**

The online version of this article (10.1007/s00259-020-04880-1) contains supplementary material, which is available to authorized users.

## Introduction

The identification of biomarkers of the pathology of Alzheimer’s disease (AD) has transformed the nature and scope of intervention trials for AD. Evidence of abnormal β-amyloid deposition in the brain by CSF or amyloid PET is now standard for inclusion of subjects in intervention trials for AD. Detection of β-amyloid pathology for secondary prevention trials in mildly symptomatic or asymptomatic subjects at risk for AD alone may be insufficient, as amyloid burden correlates poorly with the degree of cognitive impairment in AD and has limited value in estimating the lifetime risk for the development of dementia [1]. Detection of tau pathology and evidence of neurodegeneration is necessary for more complete profiling of disease stage within the Amyloid, Tau, Neurodegeneration (“A-T-N”) research framework [2]. To support more extensive phenotyping of subjects, several PET ligands for detection and quantitation of tau neurofibrillary tangles (NFTs) in brain have been developed and are being employed in clinical studies [3, 4]. T807, the [^18^F] labeled pyrido-indole discovered by Kolb et al. at Siemens, subsequently acquired by Lilly/Avid (now known as AV1451 or flortaucipir), has been the most extensively used tau PET tracer. Cross-sectional and longitudinal studies with flortaucipir are providing fundamental insights into tau pathology and progression. Nonetheless, significant off-target binding has been observed, which could confound regional estimation of tau pathology and change in signal over time [5]. Moreover, clearance of the signal from target-rich regions is slow so that relative steady state may not be reached until long after tracer injection [6] . These issues provided the motivation for developing a ligand with less off-target binding and improved clearance kinetics [7], an effort that resulted in the novel isoquinoline JNJ-64326067, which can be readily labeled with [^18^F]. In vitro, JNJ-64326067 was found to have high affinity for tau aggregates in post-mortem brain tissue from subjects with AD and high selectivity over β-amyloid aggregates. The absence of significant binding to other brain targets was tested by profiling at CEREP laboratories (https://www.eurofinsdiscoveryservices.com/), with additional testing against MAO-A and MAO-B [8] as these can confound in vivo imaging [9, 10]. Metabolism and kinetics of [^18^F] JNJ-64326067 were also evaluated in preclinical models. Displacement of fluorine by glutathione was observed in rat hepatocytes, and a signal in skull was seen in PET studies in rat. Such displacement was negligible in non-human and human hepatocytes, and no significant defluorination was predicted to occur [8]. We here report the clinical qualification of the ligand in subjects with probable AD and healthy controls including tracer kinetic modeling, biodistribution, and dosimetry.

## Methods

### Subjects and study design

The clinical evaluation of [^18^F] JNJ-64326067 was conducted in two parts, beginning with a proof-of-concept evaluation of the tracer by comparing the brain signal in subjects with probable AD to age-matched healthy subjects (study 64326067EDI0001; NCT03239561). After demonstrating that a discrete signal could be detected in brain regions known to accumulate tau aggregates in AD, a radiation dosimetry study in healthy male and female subjects was conducted (study 64326067EDI1001; NCT03581916).

Participants were recruited from a volunteer registry and response to advertisement. Healthy male and female subjects and participants with probable AD at least 50 years of age were screened for the proof-of-concept study, and healthy subjects between the ages of 18 and 75 were screened for the dosimetry study. After obtaining informed consent, all subjects underwent clinical evaluation including a T1 weighted MRI. A PET scan with [^18^F] florbetapir was obtained in proof-of-concept study subjects to determine whether they were amyloid negative (healthy controls) or positive (probable AD subjects) unless a recent amyloid PET scan was already available. Further inclusion/exclusion criteria are provided in the supplementary materials. At the request of the FDA, the first human scan was recorded as a whole-body image using a low dose of [^18^F] JNJ-64326067 (52 MBq) in a healthy female subject (aged 53) to confirm that there was no unanticipated accumulation of radioactivity in radiosensitive tissues prior to conducting further [^18^F] JNJ-64326067 PET scans. Dedicated brain scans were then obtained in 5 healthy controls and 5 participants with probable AD. On completion of the proof-of-concept study, whole-body dosimetry was measured in six subjects.

The studies were approved by the local Ethics Committee and by the FDA as part of an IND application and performed in accordance with the World Medical Association Declaration of Helsinki. Written informed consent was obtained from all participants prior to the study.

### PET tracer [^18^F] JNJ-64326067

[^18^F] JNJ-64326067 or N-[4-(^18^F) fluoro-5-methylpyridin-2-yl] isoquinolin-6-amine is a small molecule (M.W. 252.28) with selective, high-affinity binding to phosphorylated tau aggregates. It is structurally differentiated from other tau ligands currently in development. In vitro studies used for the selection of JNJ-64326067 demonstrated potent binding to aggregated tau isolated from human AD brain (inhibition constant [Ki] of 2.4 nM) and > 1000-fold selectivity over Aβ. JNJ 64326067 has an experimental Log D of 3.51 at pH 7.4; a low polar surface area of 38 Å^2^; a plasma protein binding of 93.6%, 97.0%, and 95.4%, respectively, in humans, mice, and rats; and a brain tissue binding of 98.7% [8]. Information on the radiosynthesis and production results are provided in the supplementary material.

### PET imaging and analysis proof-of-concept study

All PET scans were acquired on a Siemens Biograph PET CT camera. Each imaging session began with a CT transmission scan for attenuation correction. For dedicated brain scans dynamic PET data were acquired over two imaging sessions: 29 frames over 90 min post injection (6x 30 s, 4x 60 s, 4x 120 s, and 15x 300 s) and then 12 frames from 120 to 180 min post injection (300 s each). A break in the scan occurred between 90 and 120 min. Venous blood samples were collected post injection at 5, 10, 30, 60, and 90 min for measurement of whole blood and plasma radioactivity, intact tracer fraction by HPLC, and plasma free fraction. Additional information on the metabolite analysis is provided in the supplementary material.

Raw image data were reconstructed using OSEM (4 iterations, 16 subsets) and corrected for attenuation, randoms, scatter, and dead time. Reconstructed PET imaging data volumes were processed with PMOD version 3.802 (PMOD Technologies, Zurich, Switzerland), where the images were motion and decay corrected, coregistered with the subject’s T1 MRI, and subsequently normalized into MNI (Montreal Neurological Institute) space. The subject’s MRI had been segmented into gray matter, white matter, and CSF maps. Volumes of interest (VOIs) from the Hammers atlas were projected onto the image data [11, 12]. Average activity concentration (kBq/cm^3^) within each VOI, constrained to gray matter voxels for cortical regions, were determined, and time activity curves (TACs) were generated. TACs and PET images were expressed in SUV units (g/mL) by normalizing to the weight of the subject and the injected dose. The ventral cerebellar cortex was used as a reference region for normalizing activity in target regions to generate SUVr values. SUVr images were computed between 60 and 90 min and 120–180 min. To assess differences in [^18^F] JNJ-64326067 binding between participants with AD and age-matched healthy control (HC), SUVr and distribution volume ratio (DVR) were used as outcome measures. SUVr TACs were averaged between 30–60, 60–90, 120–140 min, and 120–180 min. DVR was estimated by non-invasive Logan graphical analysis (LGA) [13] using 90 min or 180 min of image data with fit starting time (t*) at 25 min, also using ventral cerebellar cortex as a reference region. The tissue to plasma clearance rate, *k*_2_′, was determined as an average across VOIs of *k*_2_′ derived from the Simplified Reference Tissue Model [14]. SUVr over different time intervals and DVR were compared via linear regression analysis. The differences between AD and HC in SUVr and DVR for each region were assessed with *t* tests for independent groups with unequal variances, and Cohen’s d effect size estimates.

### PET imaging and analysis: Dosimetry study

The 6 dosimetry subjects received a bolus intravenous administration of 318 ± 53 MBq of [^18^F] JNJ-64326067 followed by a series of whole-body PET image acquisitions consisting of 9 bed positions from the vertex of the head to the thighs over a period of up to approximately 5.5 h in three scanning sessions. A total of 9 whole-body scans (or passes) were acquired over 3 sessions. The scanning sessions were separated by ~ 30 min breaks during which the subjects could leave the scanner bed and during which urine was collected for measurement of radioactivity.

The Organ Level Internal Dose Assessment (OLINDA) 2.0 software package (Hermes Medical Solutions) was used to estimate the organ and whole-body radiation absorbed doses. Complete information on the scanning protocol and methods of analysis is provided in the supplementary material.

## Results

### [^18^F] JNJ-64326067 in brain

#### Subjects

The mean age of AD subjects was 73 (range 63–85) years, and the mean age of the HC was 69.3 (range 60–75) years. Demographic and dose data for subjects in the proof-of-concept study are summarized in Table 1. All HC subjects were amyloid negative, and all AD subjects were amyloid positive by visual read of [^18^F] florbetapir PET scans.

**Table 1:** Demographics of AD subjects and controls for dedicated [^18^F] JNJ-64326067 brain scans. Numbering of subjects reflects the order of enrollment in to the study and does not include screen failures or the initial HV who underwent the initial low-dose (52 MBq/1.40 mCi [^18^F] JNJ-64326067) whole-body scan. Results of florbetapir scans are from visual reads

Subject number	Cohort	Age	Sex	Florbetapir scan	MMSE	CDR	ADAS-Cog	[^18^F] JNJ-64326067 Dose (MBq)	JNJ-64326067 mass (μg)
4	AD	77	M	Pos	26	0.5	22	335.59	2.21
6	AD	63	M	Pos	21	0.5	19	335.22	0.59
8	AD	85	M	Pos	12	2	16	347.43	2.28
11	AD	74	M	Pos	14	1	36	337.44	3.14
14	AD	66	F	Pos	23	1	29	312.65	1.74
5	HV	75	F	Neg	28	0	6	321.9	2.31
9	HV	60	F	Neg	29	0	4	347.06	1.57
10	HV	73	M	Neg	28	0	6	349.65	2.12
13	HV	71	M	Neg	28	0	11	337.81	2.05
15	HV	78	M	Neg	29	0	6	342.99	1.30

Age, administered dose of [^18^F] JNJ-64326067, and JNJ-64326067 mass per dose were not significantly different between AD and HV subjects (*t* test)

*MMSE* Mini Mental Status Exam, *CDR* Clinical Dementia Rating Scale, *ADAS-Cog* Alzheimer’s Disease Assessment Scale-Cognition

**Fig. 1:**
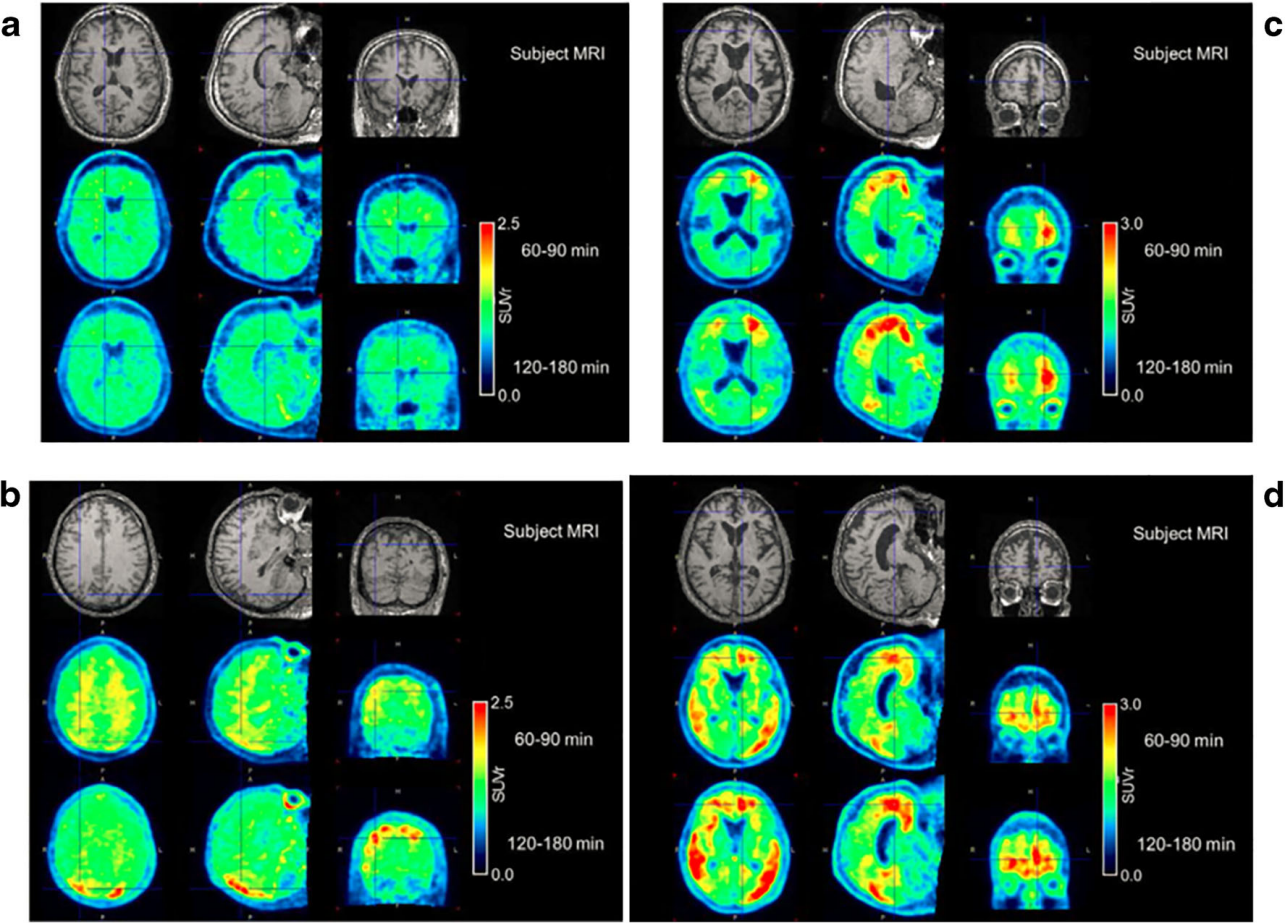
Individual MRI (top), 60–90 min (middle), and 120–180 min (bottom). PET SUVr images from HV Subjects 13 (**a**), and 10 (**b**), and AD subjects 6 (**c**) and 11 (**d**). Signal in the occipital cortex in subject 10 underlie the location of pellets in the skull from a shotgun accident as a youth

Visual assessment of the 120–180 min scans: Healthy subjects in the proof-of concept study displayed low brain retention (e.g**.,** Fig. 1a) with the exception of one subject, a 73 year-old male who had focal retention in the occipital and parietal lobe (HC 10). Calcified artifacts in the occipital region of the scalp and skull overlying the regions of focal retention were visible on the CT scan, attributable to a hunting accident with a shotgun he had experienced as a youth (Fig. 1b). Four of the 5 AD subjects had visible retention in the temporal cortex, one AD subject had relatively more retention in the frontal cortex (Fig. 1c), and one AD subject had no visible retention (AD 04) (Figure 2S in supplementary material). There was no evidence of PET signal in the skull suggestive of bone uptake of [^18^F] fluorine (Figure 4S in supplementary material).

#### Quantitative analysis

Significant within frame motion occurred between 120 and 180 min in one AD subject (AD 8) that precluded inclusion of these data for reliable quantitative analysis. Significant between frame motion occurred in another subject (AD 14) requiring censoring of frames before 65 min and after 165 min (see Figure 6S in supplementary material).

**Fig. 2:**
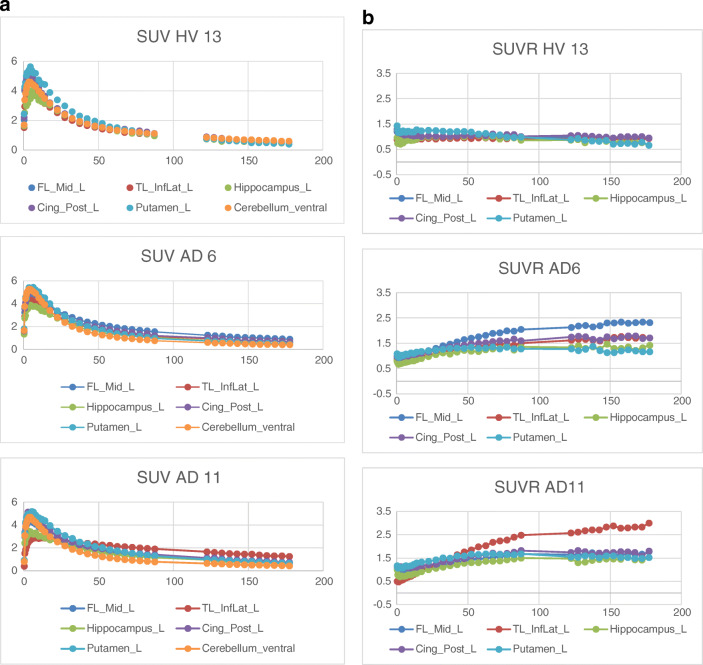
**a** Representative [^18^F] JNJ-64326067 SUV time-activity curves HV and AD subjects. **b** [^18^F]JNJ-64326067 SUVr time-activity curves from the same HV and AD subjects. Abbreviations: medial frontal cortex (FL_Mid_L), inferior lateral temporal cortex (TL_InfLat_L), posterior cingulate (Cing_Post_L)

In general, uptake of the tracer into brain was rapid with peak SUVs between 5 and 10 min, followed by moderately rapid clearance (Fig. 2a). Brain regional SUV normalized to cerebellum (SUVr) TACs in HC showed little change by 60 min post injection. The regional SUVr TACs in 4 AD subjects continued to rise through 90 min post injection and approached a plateau in most cortical regions by the beginning of the 120 to 180 min acquisition period (Fig. 2b). The AD subject with no visible retention (AD 04) had no regional increases in SUVr after 60 min (Table 1S and Figure 6S in supplementary material). By the end of the scan (up to 180 min) three of the AD subjects showed high retention in the temporal cortex (up to SUVr of 3.1 in right inferolateral temporal in one subject); one AD subject showed high retention in inferotemporal cortex (SUVr 1.7 on the right and 1.68 on the left) and higher retention in the left medial frontal cortex (SUVr of 2.2) (Table 2S in supplementary material). The tracer was rapidly metabolized in the periphery with 75 ± 10% and 58 ± 14% (mean ± SD) of the parent fraction remaining at 5 min in HC and AD subjects (respectively), 45 ± 11% and 36 ± 13% at 10 min in HC and AD, 17 ± 6% and 12 ± 11% at 30 min in HC and AD, and 9 ± 4% and 6 ± 3% at 60 min in HC and AD post injection. The free fraction of parent compound in plasma, measured by ultrafiltration, was 0.80 ± 0.14% in HC and 0.77 ± 0.43% in AD subjects (Figure 1S in supplementary material).

**Fig. 3:**
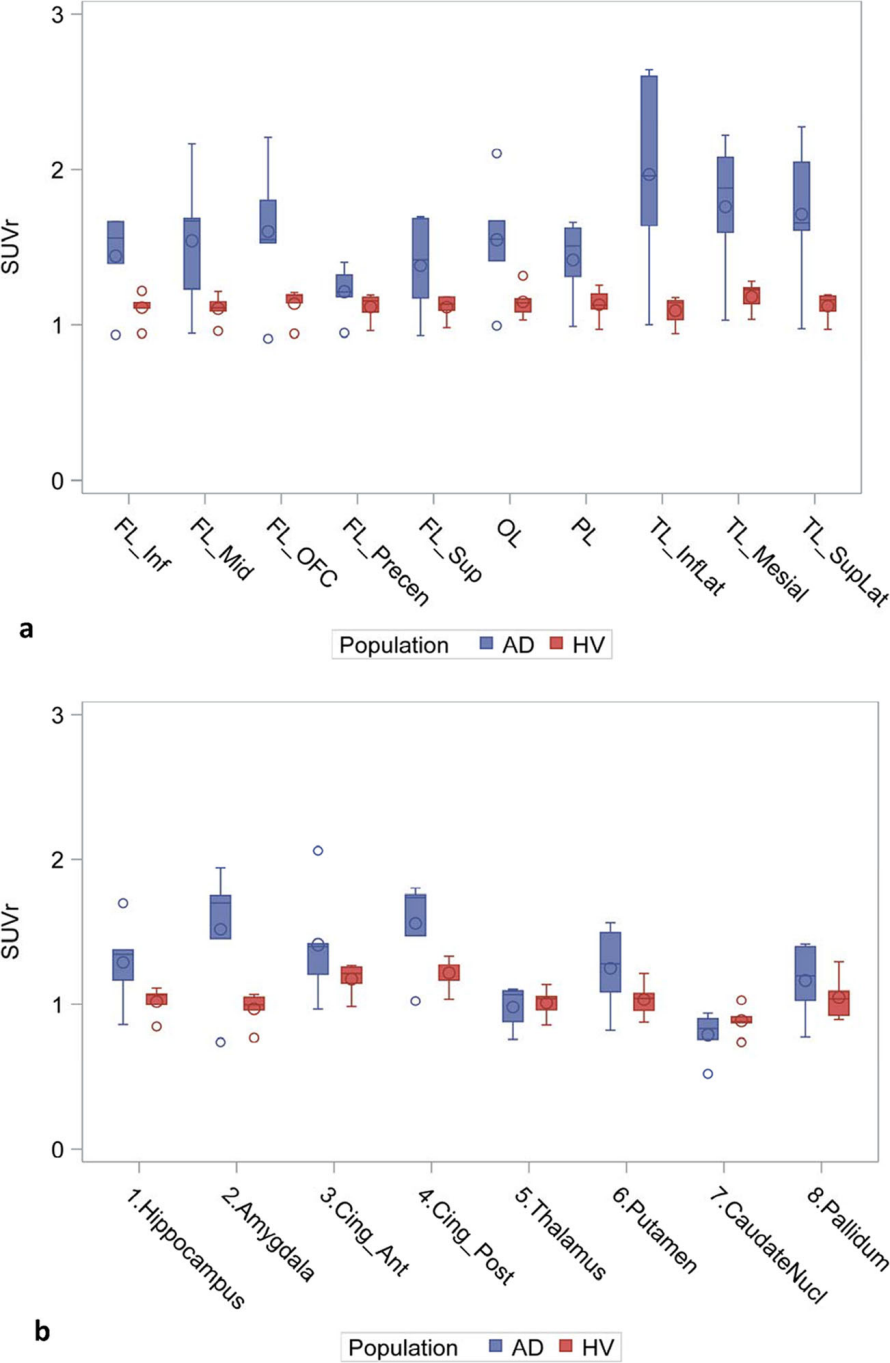
Regional SUVr values for all AD and HV subjects: cortical (**a**) and subcortical and mesial regions (**b**). FL frontal, OL occipital, TL temporal, Cing cingulate. Box represents the interquartile range (IQR); i.e., 25th–75th percentiles. Inside the box: median (horizontal line), mean (circle). Whiskers cover the data within 1.5 IQR. Circles outside the whiskers show the observations beyond the reach of the whiskers. Results for right and left were similar; left regions only are depicted for clarity

**Fig. 4:**
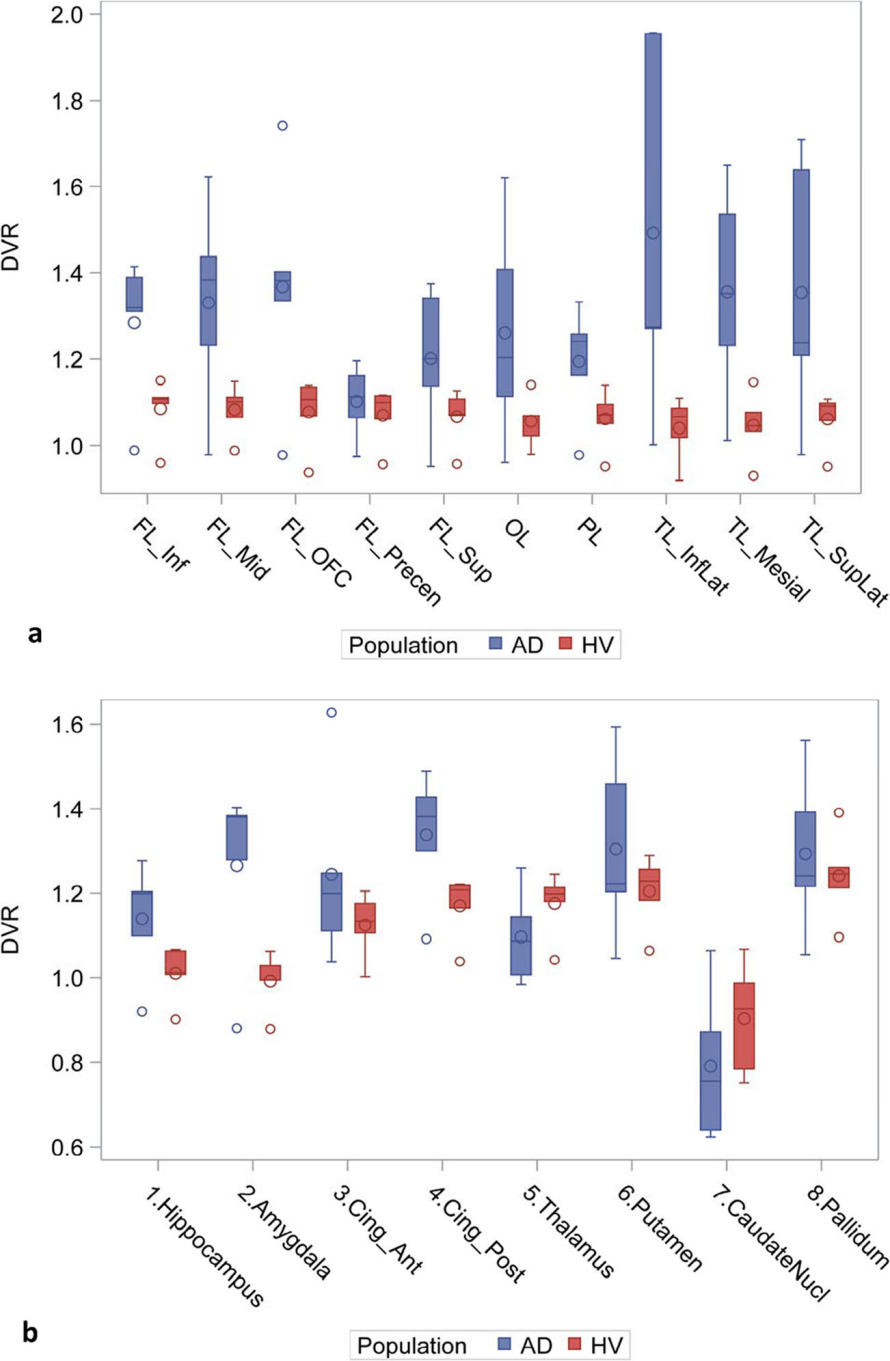
Regional DVR values for all AD and HV subjects: cortical (**a**) and subcortical and mesial regions (**b**). Abbreviations are the same as in Fig. 3. Results for right and left were similar; left regions only are depicted for clarity

Figure 3 compares the range of SUVr values in each subject group by left cortical (Fig. 3a) and subcortical and mesial regions (Fig. 3b) for the 120 to 140 min time frame, which was selected as a period of stability of the cortical signal in AD and feasible duration for late acquisition scans. Temporal regions had the highest cortical SUVr values in AD subjects followed by frontal and parietal regions with effect sizes for the difference between AD and HC (Cohen’s d) up to 1.78 (Table 2aS in supplementary material). Amygdala had the highest mean SUVr in AD subjects among the mesial regions. Among the subcortical regions, thalamus had low signal and did not differ between AD and HC (Fig. 3b). Putamen and pallidum were modestly elevated in AD compared to HC with effect sizes of 0.93 and 0.52, respectively (Table 2bS in supplementary material). Figure 4 provides the same comparison for regional DVR values by subject group and showed a similar pattern. The effect sizes for DVR were also similar to those for SUVr (Tables 3aS and 3bS in supplementary material).

**Fig. 5:**
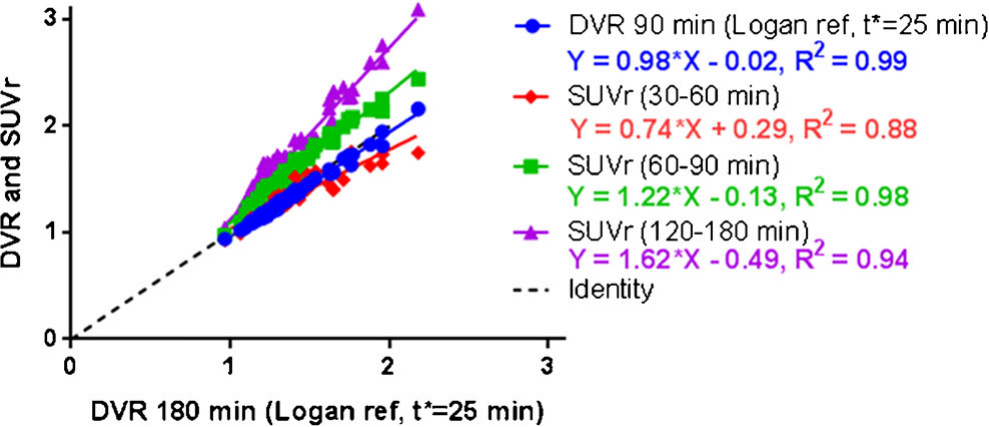
Correlation of [^18^F] JNJ-64326067 DVR using 90 min of data, SUVr averaged between 30 and 60, 60–90, and 120–180 min with DVR using 180 min of data. DVR was estimated using LGA. The ventral cerebellum reference region was used for both SUVr and DVR. These regressions were done using regional values in AD subjects 06, 11, and 14

Regional DVR values in 3 AD subjects with higher retention in temporal cortex with acceptable image quality over time (AD subjects 6, 11, and 14) were further explored and compared to SUVr values by linear regression and Bland–Altman plots. Regional DVR over 90 min had excellent agreement with DVR over 180 min (*R*^2^ = 0.99). Average SUVr over 60–90 min and over 120–180 min overestimated signal relative to DVR but were highly correlated with DVR over 180 min. Average SUVr over 30–60 min underestimated signal relative DVR (Fig. 5).

### Dosimetry

Whole-body dosimetry was measured in six subjects (3 males and 3 females), having a mean age of 66.8 (range 54–75) years.

The target organ with highest exposure (critical organ) was found to be the right colon (ascending +1/2 transversal colon) for male subjects, which received a dose of 0.152 ± 0.0548 mSv/MBq, and urinary bladder wall for females, which received a dose of 0.185 ± 0.0328 mSv/MBq. The average whole-body effective dose, ED, calculated with ICRP-103 tissue weighting factors was determined to be 0.0254 ± 0.0035 mSv/MBq for ICRP-89 adult male and 0.0257 ± 0.0011 mSv/MBq for ICRP-89 adult female. The average ED from all subjects was determined to be 0.0255 ± 0.0023 mSv/MBq, or 4.72 ± 0.43 mSv per 185 MBq tracer injection, which is comparable to other [^18^F] labeled PET tracers. The values for each of the organs and tissues and ED values by subject are provided in the supplementary materials.

#### Safety

There were no clinically significant changes in vital signs, clinical laboratory values, or ECGs in any of the subjects during the study. Adverse events were mild, none were attributed to exposure to the tracer dose, and a few were related to study procedures such as use of intravenous lines or the need to limit movement during the scan.

## Discussion

This was the first clinical evaluation of [^18^F] JNJ-64326067, a high-affinity PET ligand for tau aggregates associated with Alzheimer’s disease, which displays high selectivity over β-amyloid aggregates, absence of affinity for other brain targets especially monoamine oxidase, and suitable metabolic and pharmacokinetic profiles. Radiolabeling was robust and reliable, and all productions met release specifications. Brain uptake was excellent with a peak SUV of 5–10 g/mL. Clearance from non-target regions was rapid and retention appeared to plateau in regions known to accumulate tau aggregates by 120 min post injection. There was no evidence of bone uptake that had been seen in rat, consistent with the preclinical studies which differentiated metabolism in primates [8].

This proof-of-concept study included a very limited number of subjects as it was intended to determine whether further development and testing were warranted. All AD subjects were amyloid positive by florbetapir PET with cortical signal evident throughout the cerebrum. Determination of whether specific retention of [^18^F] JNJ-64326067 occurred in regions with probable tau pathology relied on reference tissue methods and did not include arterial sampling that would have supported compartmental modeling. The cerebellum has been evaluated extensively as a reference region for tau PET tracers and is supported by the absence of any nonpolar metabolites in preclinical studies [8]. As a proof-of-concept, we first wanted to see how [^18^F] JNJ-64326067 performed compared to tau PET tracers that use reference tissue for quantitation of the NFT signal as a readily implemented and tolerated protocol for clinical trials in AD [6, 15] while not burdening subjects with invasive sampling. Three of the five subjects with AD exhibited high retention of [^18^F] JNJ-64326067 signal in temporal cortical regions classically associated with tau pathology in AD (subjects 6, 11, 14), one showed high retention in left frontal lobe (subject 8) in addition to the temporal cortex, while one AD subject [4] exhibited no retention of signal. This subject was 77 years old, the median age of onset of hippocampal sclerosis. Hippocampal sclerosis presents with amnestic impairment, and patients are commonly diagnosed with AD dementia ante mortem. Subjects found to have hippocampal sclerosis at autopsy can be amyloid PET positive and tau PET negative [16]. Interestingly, this AD subject with no apparent cortical tau signal had the least cognitive impairment (MMSE of 26), while the AD subject with the highest cortical signal (subject 11) had the greatest cognitive impairment (MMSE of 14) (Table 1).

Off-target retention was visually absent in choroid plexus or meninges in contrast to what has been seen with other tracers [5, 9]. No visual or significant visual or measured signal was seen in thalamus in either AD or HC subjects suggesting that selection against affinity for MAO-B was successful. No visual and negligible measured signal was observed in caudate, but the measured signal was slightly elevated in putamen and pallidum, especially in AD subjects. Neurofibrillary tangles have been observed throughout the striatum, notably the ventral or ‘limbic’ striatum [17]. Iron or ferritin have been identified as potential causes of off-target binding [3, 5], and both putamen and pallidum have notably high concentrations of iron [18] although iron concentrations might not be expected to differ between AD and HC. The punctate regions of retention in occipital and parietal cortex in the healthy subject who had experienced a shot gun accident as a youth may potentially indicate tau pathology secondary to the injury [19, 20], although off-target binding cannot be ruled out. Full characterization of possible off-target binding will require a larger number of subjects, including a broader age range, as well as autopsy confirmation.

In addition to reducing off-target binding, JNJ-64326067 was also selected for fast clearance from non-target tissue. Clearance from brain in healthy subjects was as rapid as was seen in non-human primate [8]. Clearance from target regions was slow, with SUVr still rising in the highest signal regions after 90 min and appearing to approach an asymptote only after 120 to 150 min post injection. This is similar to the kinetics reported for flortaucipir and MK-6240 [6, 15] and may be typical of high-affinity ligands for NFTs. The [^18^F] amyloid PET tracers also typically achieve quasi-steady state in target rich areas long after injection [21, 22]. Amyloid and tau aggregates in human likely present high-density binding environments allowing rapid rebinding of ligand, delaying clearance of the ligand, and achieving quasi-steady state. As NFTs are intracellular, the neuronal membrane may further delay diffusion from the target [23]. This suggests that timing of acquisition scans may need to be adjusted to late after tracer injection in subjects who are anticipated to have regions with high NFT burden or would be expected to develop high NFT burden over time. Only a limited number of subjects with mild to moderate disease were evaluated in this study. Subjects with more advanced or advancing tau pathology may take longer to approach an asymptote and speak to the need to evaluate this tracer in a larger sample.

Tracer elimination occurred via both the hepatobiliary and urinary system, causing the gallbladder, intestines, and urinary bladder to be main source organs for radiation burden. The average whole-body effective dose (ED) was calculated to be 0.0257 mSv/MBq for adult females and 0.0254 mSv/MBq for adult males, which compares favorably to other [^18^F] radiopharmaceuticals [24]. According to the US federal regulations specified in Title 21 CFR 361.1 the radiation doses to the whole body, gonads, active blood-forming organs, and lens of the eyes should not exceed 50 mSv annually and 30 mSv for a single study. The dose absorbed by all other organs should not exceed 150 mSv annually and 50 mSv per study [25]. Based on the critical organ doses and the whole-body effective doses the total number of scans at an injected dose of 185 MBq (5 mCi) would conservatively be limited to 4 per year in the USA, and around 2 per years in the EU and regions following the ICRP recommendations [26] .

**Fig. 6:**
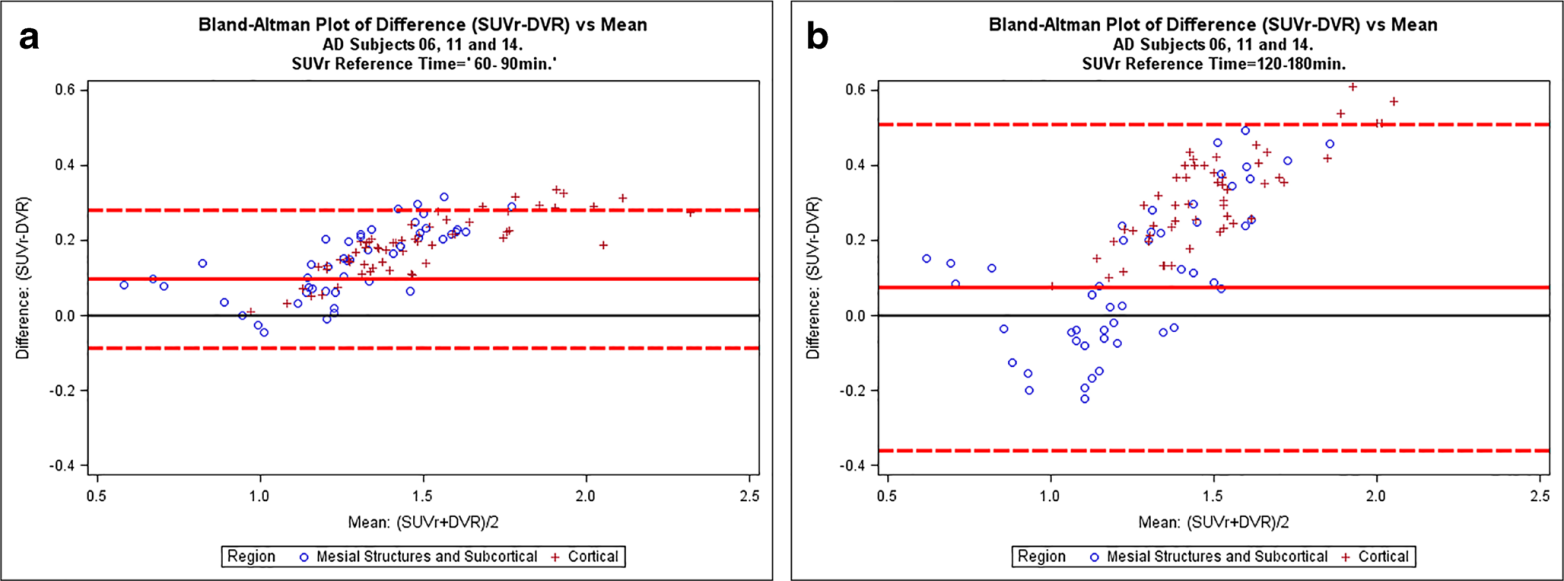
Bland–Altman plots of SUVr vs. DVR. Bland–Altman plots of SUVr vs. DVR for **a** 60–90 min and **b** 120–180 min for AD subjects 06, 11, and 14. Each point shows a pair of (SUVr, DVR) measures taken in the same subject × region. The *x*-coordinate is the average of (SUVr, DVR), and the *y*-coordinate is the difference (SUVr-DVR). The black horizontal line through *y* = 0: reference where SUVr = DVR. Above this line SUVr>DVR, and vice versa. The red horizontal line represents the trough of the mean of all difference observations. The red dotted lines indicate the 95% threshold for the differences (i.e., mean ± 2SD)

In the three AD subjects who showed high retention of [^18^F] JNJ-64326067 in temporal regions, 90 min of DVR data were highly correlated with 180 min of data suggesting that a shorter acquisition period could be used for dynamic studies. SUVr (30–60 min) underestimated DVR, while SUVr (60–90 min) and SUVr (120–180 min) overestimated signal relative to DVR similar to observations at quasi steady state with amyloid [27] and other tau PET ligands [28]. The SUVr (120–140 min) range in those AD subjects across regions was similar to the range of values reported for AV1451 [29] suggesting that [^18^F] JNJ-64326067 may have a comparable dynamic range. The 120–140 min time frame was chosen for the group comparison of SUVr values, as the cortical signal in AD subjects appeared have plateaued and as a practical duration for late acquisition “static” scans. The Bland–Altman plots over both the 60–90 min and 120–180 min intervals indicated that variability between SUVr and DVR increased as a function of the magnitude of the signal (Fig. 6). The range of variability in the Bland–Altman plots was substantially less for the 60–90 min period, and while overestimation of the signal may occur, this period may be more reliable for quantitation of SUVr. Defining an optimal acquisition period will require evaluation in a larger number and more diverse range of subjects. Complete characterization of the kinetics of [^18^F] JNJ-64326067 will require inclusion of arterial sampling. Compartmental modeling from an arterial input function may not assist in identifying off-target binding [30]; however, this would be of value as a gold standard comparator for DVR, SUVr, and other reference tissue methods; selection of an optimal reference region at least for cross-sectional studies; and to determine whether the fractional blood volume contributes to the regional cerebellar signal [31]. The larger effect sizes seen with DVR values and potential for obtaining blood flow data such as R1 images or analysis of the initial image frames speak to the value of dynamic scans and support their use if time and PET center staffing allows. The acquisition protocol can be adapted to allow for breaks for subject comfort and tolerability, as was done in this study.

These results support [^18^F] JNJ-64326067 as a novel isoquinoline PET tracer for the detection and quantitation of tau aggregates in Alzheimer’s disease research, joining the armamentarium of imaging biomarkers for detection of tau pathology. Tau PET imaging may aid in the diagnosis and staging of cognitive disorders, monitoring of disease progression, and might serve as a biomarker of therapeutic effect for interventions targeting tau. Further characterization and validation of this tracer will require additional clinical studies to determine the optimal acquisition time and reference region, off-target binding, test-retest variability, the dynamic range with disease severity, and the magnitude of longitudinal changes, as well as the suitability of this tracer for earlier disease stages and other tauopathies.

## Electronic supplementary material

ESM 1 (DOCX 6986 kb).
