# Supplementary material for: Clinical evaluation of [18F] JNJ-64326067, a novel candidate PET tracer for the detection of tau pathology in Alzheimer’s disease
Source: Eur J Nucl Med Mol Imaging. 2020 Jun 13;47(13):3176–85. doi: 10.1007/s00259-020-04880-1 (PMC7680304; doi:10.1007/s00259-020-04880-1)
Supplement: Supplementary file 1 — (DOCX 6986 kb). [file 259_2020_4880_MOESM1_ESM.docx]

# Inclusion Criteria (for all subjects)

- Written informed consent must be obtained before any assessment is performed.
- Female subjects must be documented by medical records or physician’s note to be either surgically sterile (by means of hysterectomy, bilateral oophorectomy, or tubal ligation) or post-menopausal for at least 1 year. Male subjects and their partners of childbearing potential must commit to the use of two methods of contraception, one of which is a barrier method for male subjects for the study duration.
- Male subjects must not donate sperm during the study and for 3 months after completion.
- The subjects must be willing and able to cooperate with study procedures.

## Inclusion Criteria for Healthy Subjects

- Males and females aged ≥50 years. Healthy with no clinically relevant finding on physical examination at screening and upon reporting for the [^18^F] JNJ-64326067 imaging visit.
- No cognitive impairment from neuropsychological assessments as judged by the investigator.
- Have screening [18F] florbetapir PET imaging demonstrating no significant amyloid binding based on qualitative analysis (visual read).
- No family history of Alzheimer’s disease or neurological disease associated with dementia
- Have a CDR score=0

## Inclusion Criteria for Subjects with a Diagnosis of Probable Alzheimer’s Disease (AD)

- Males and females aged 50 to 90 years.
- Have probable Alzheimer's disease, based on the NINCDS/ADRDA and DSM-IV
- criteria.
- Have a CDR score of 0.5 or greater at screening.
- Have an MMSE score ≤ 28.
- Have screening [^18^F] florbetapir or prior amyloid (in the last 12 months) PET imaging demonstrating amyloid binding based on qualitative (visual read).
- A brain MRI that supports a diagnosis of AD, with no evidence of focal disease to account for dementia or MRI exclusion criteria.
- Medications taken for symptomatic treatment of AD must be maintained on a stable dosage regimen for at least 30 days before the screening visit.
- Signed and dated written informed consent or assent obtained from the subject and the subject’s legally authorized representative or caregiver (if applicable).
- The subject has an appropriate caregiver capable of accompanying subject, if necessary.

## Exclusion Criteria (for all subjects)

Subjects fulfilling any of the following criteria are not eligible for inclusion in this study:

- Current or prior history (within the past 6 months) of any alcohol or drug abuse.
- Laboratory tests with clinically significant abnormalities and/or clinically significant unstable medical illness.
- Subject has received an investigational drug or device within 30 days of screening.
- Prior participation in other research protocols or clinical care in the last year in addition to the radiation exposure expected from participation in this clinical study, such that radiation exposure exceeds the effective dose of 50 mSv, which would be above the acceptable annual limit established by the US Federal Guidelines.
- Pregnancy, lactating or breastfeeding.
- Women of childbearing potential.
- Evidence of clinically significant gastrointestinal, cardiovascular, hepatic, renal, hematological, neoplastic, endocrine, alternative neurological, immunodeficiency, pulmonary, or other disorder or disease.
- Unsuitable veins for repeated venipuncture.
- MRI exclusion criteria include: evidence of cerebrovascular disease (more than two lacunar infarcts, any territorial infarct >1cm3, or deep white matter abnormality corresponding to an overall Fazekas scale of 3 with at least one confluent hyperintense lesion on the FLAIR sequence that is ≥ 20 mm in any dimension), infectious disease, space-occupying lesions, normal pressure hydrocephalus or any other abnormalities associated with CNS disease.
- Implants such as implanted cardiac pacemakers or defibrillators, insulin pumps, cochlear implants, metallic ocular foreign body, implanted neural stimulators, CNS aneurysm clips and other medical implants that have not been certified for MRI, or history of claustrophobia in MRI.

## Exclusion Criteria for Subjects with Probable AD

- Has received treatment that targeted amyloid-β or tau within the last 3 months.

Unique criteria for the dosimetry study:

- Male or female subject 18 to 75 years of age, inclusive. Women must be post-menopausal.

# Radiosynthesis

The tracer was synthesized by nucleophilic substitution reaction of the NO2-group on the precursor molecule (JNJ-64515685). [^18^F] fluoride was eluted into a solution of potassium carbonate and Kryptofix-222, evaporated by heating and then a solution of the precursor in anhydrous dimethylsulfoxide was added followed by heating; the reaction was carried out in a TRACERlab® (GE Healthcare). Purification was performed by semi-preparative HPLC, submitted for analysis and determination that dose and release specifications were met. The drug product for injection consisted of [^18^F] JNJ-64326067 dissolved in a sterile solution of normal saline containing approximately 3.3% (v/v) ethanol (EtOH) and 0.47% (w/v) sodium ascorbate. The drug product was sterile filtered into a sterile, filter-vented vial pre-loaded with 15 mL of normal saline.

Step 1

[^18^F] fluoride was transferred from the source vial and trapped on an ion exchange cartridge. It was then eluted with a solution of potassium carbonate and Kryptofix-222 into the reaction vessel (RV1) of the TRACERlab® module. The solution was evaporated by heating at 95°C for 4 min under vacuum and helium flow. Acetonitrile (1 mL) was added to RV1 and evaporation continued under the same conditions for 2 min. After a second addition of acetonitrile (1 mL), final evaporation was carried out at 95°C for 2 min under vacuum and helium flow. The reactor was then cooled to 60°C.

Step 2

A solution of the precursor in anhydrous dimethylsulfoxide was added to the reaction vessel. The reaction mixture was heated at 160°C for 10 min.

Step 3

The reactor was cooled to 40°C and the contents are diluted with HPLC eluent and the mixture was transferred into the loop-loading vials (RV2). The reactor was rinsed with water for injection (WFI) and the rinse was transferred into RV2. The contents of RV2 are transferred into the HPLC injector loop for purification.

Step 4

Purification was performed by HPLC using a semi-preparative Agilent Eclipse XDB C18 column (5 μm, 250 x 9.4 mm) column eluted with a mixture of acetonitrile/ammonium acetate (10 mM), 45/55, v/v, at 4 mL/min.

# Metabolite Analyses and Free Fraction:

The metabolite analysis was performed using a HPLC system consisting of a Phenomenex Luna C18(2) (10 x 250 mm) column eluted with a mobile phase consisted of a mixture of acetonitrile and water with 0.2% of triethylamine in a 70/30 ratio at a flow rate of 4 mL/min. The detection was performed using a gamma detector (Posi-Ram or Gamma-Ram, LabLogic, Brandon, Florida). Prior to the analysis of plasma samples, a sample containing ~0.5 μCi of radiotracer in ~2 mL of a 50/50 mixture of acetonitrile and water was injected on the HPLC to identify the retention time of the parent compound.

Fraction of unchanged radiotracer in plasma was determined by HPLC in selected time points. Plasma samples were processed by acetonitrile denaturation, treating 1 mL of plasma with 1 mL of acetonitrile. After vigorous mixing and centrifugation at 3,000 g for 10 min, the supernatant was transferred to an autosampler vial and injected onto HPLC. Sample vials and pellets were counted in the gamma counter for 1 min to calculate the extraction percentage. Gamma chromatograms were analyzed by integration of all radioactive peaks. The percentage of parent compound was calculated by dividing the area under the peak representing parent compound by the sum of the area of all radioactive peaks.

For the determination of plasma free fraction (the fraction of radioligand that is not protein bound, f_p_), 200 μL aliquots of plasma spiked with the radiotracer (~2 μCi for 1 mL of plasma) were pipetted in duplicates into ultrafiltration units (Amicon Centrifree 30, Millipore) and centrifuged at 20°C for 20 min at 3,000 g. The radioactivities of the ultrafiltrate (50 μL aliquot) and the filtration unit were counted, repeated in duplicate. The plasma free fraction f_p_ was calculated as the ratio of the ultrafiltrate activity concentration to the plasma activity concentration and expressed as the average of the two measurements.

Figure 1S. [^18^F] JNJ-64326067 parent fraction in venous plasma over time for HV and AD subjects.

# Radiolabeling results:

Eleven productions were used in the study with an average radiochemical yield of 40.8 ± 9.8% (maximum yield 55.6% and minimum yield 28.7%). Average end of synthesis activity was 5.8 ± 1.7 mCi/µmol (range 3.3 to 9.3 mCi/µmol). The release specification for mass was ≤ 5 µg/ml; average mass over the 11 productions was 0.36 ± 0.16 µg/ml (range 0.12-0.79 µg/ml). All productions met specified limits for pyrogen (LAL), Kryptofix, ethanol, and residual solvent content and passed sterility testing.

# Dosimetry

Whole body imaging studies were performed on a Siemens Biograph PET CT camera to determine the biodistribution of [^18^F] JNJ-64326067 and to estimate radiation absorbed doses to source organs and whole body. Subjects received a bolus intravenous administration of 318.4 ± 53.3 MBq (8.6 ± 1.4 mCi) of [^18^F] JNJ-64326067 followed by a series of whole body PET image acquisitions consisting of 9 bed positions from the vertex of the head to the thighs over a period of up to approximately 5.5 hours in three scanning sessions. The scanning sessions were separated by ~30 min breaks during which the subjects could leave the scanner bed. The first scanning session included 5 whole body passes (2×60, and 3×120 seconds per bed position). The second and third session included 2 whole body passes each (2×270 seconds per bed position). A whole-body CT transmission scan was acquired prior to each imaging session. Urine collection was performed 3-5 times, 1-2 times after each scanning session, and up to 6 hours post radiotracer injection to measure the excretion of [^18^F] JNJ-64326067 through the urinary tract. Radioactivity in urine was measured and converted to percentage of injected activity ( %ID).

Whole body tomographic PET images were analyzed in PMOD 3.8 (PMOD Technologies, Zurich, Switzerland). Volumes of interest (VOI) were manually delineated to encompass the organs visually identified as source organs. VOIs were shifted as needed, mostly in the bed plane and between imaging sessions to accommodate for patient movement between acquisitions. Activity within these volumes of interest is expressed in units of total radioactivity (kBq). Radioactivity was corrected for body attenuation, but not for decay, and time activity data (TAC, kBq) generated for each source organ. Source organs TACs were then expressed as %ID (percent injected dose) by normalization to the injected activity. Organ number of disintegrations (i.e. kinetic values), in units of MBq · h MBq^-1^ were computed from area under the non-decay corrected TACs via trapezoid method. Area under the curve from end of imaging to infinity was computed with the assumption of physical decay only following the last imaging time point. The ICRP 100 HAT gastrointestinal (GI) tract model (1) was applied to compute kinetic values in the left, right, and sigmoid (i.e. rectum) colon and small intestine, with the assumption that activity enters the GI tract via the small intestine (no absorption from either intestine or colon). The intestinal decay corrected time-activity curve was used for estimation of the fraction of the radioactivity entering the intestine during the imaging period. A gallbladder emptying model was employed and gallbladder kinetic values were computed based on the model assumptions. The gallbladder filling for all subjects was modeled in GraphPad software with a plateau plus exponential function

$$y=\left\{ \begin{aligned} 0, &x<x_{0} \\ Plateau*\left( 1-\exp\left( -k*\left( x-x_{0} \right) \right) \right), &x\geq x_{0} \end{aligned} \right.$$

by fitting the decay corrected gallbladder time activity curves over the filling period. The fitted data was further modeled for bile ejection based on data for Ensure Plus (Abbott Laboratories), a commercially available lactose-free fatty-meal food supplement, which causes gallbladder contraction. A normal gallbladder ejection fraction (GBEF) of 60% was used, based on the literature data (2). GBEF, typically measured with cholescintigraphy, is defined as a percentage decrease in gallbladder activity following the administration of the stimulant. The gallbladder emptying was modeled as occurring at a time defined as the ejection time (Tej), with 0 latency, and described as an exponential decrease by 60 % (i.e. GBEF) over a 60 min interval, followed by a plateau to the end of imaging. The number of disintegrations in the gallbladder and the absorbed doses were calculated as per the procedure described in this report. An ejection time of 60 min p.i. was used. Modeling was performed in Matlab (MathWorks, Inc.).

The urinary bladder (UB) excretion was modeled and the kinetic values calculated by fitting an exponential to the combined cumulative urinary bladder imaging data with the measured urine samples collected after each of the 3 scanning sessions. Parameters representing the fraction leaving the body via urine and biological half-time were obtained from the fit and used for modeling UB voiding for all subjects. Urinary bladder voiding models with voiding intervals of 2.0 hours were applied. Urine samples were collected before injection, after the first break (2 hours after injection time), second break (4 hours after injection time) and at the end of the study (5.5 hours after injection time). Volume of collected samples was measured. Triplicate aliquots of 200 μL of each sample of urine were counted in the gamma counter for 1 min (400-1400 keV window).

Organ Level Internal Dose Assessment (OLINDA) 2.0 software package (Hermes Medical Solutions) was used to estimate the organ and whole-body radiation absorbed doses. OLINDA uses Medical Internal Radiation Dose (MIRD) methodology (3). "NURBs" ICRP-89 adult male (73 kg) model was used to calculate the referent s-factors (4). Tissue weighting factors defined in the ICRP Publication 103, 2007 (5) were used to calculate the whole body effective dose (ED). Legacy ED values based on ICRP-60 were also provided. All other MIRD assumptions about the homogeneity of source organ distribution were employed.

# Supplemental Data

**Table 1S:** [^18^F] JNJ-64326067 SUVr averaged between 30 and 60 min in HV and AD subjects. FL=frontal, OFC=orbital frontal, OL=occipital, PL=parietal, TL=temporal.

| **Region** | **HV** | | | | | **AD** | | | | |
| --- | --- | --- | --- | --- | --- | --- | --- | --- | --- | --- |
|  | **05** | **09** | **10** | **13** | **15** | **04** | **06** | **08** | **11** | **14** |
| FL_OFC_L | 1.15 | 1.15 | 1.17 | 0.94 | 1.51 | 1.10 | 1.37 | 1.33 | 1.66 | 1.35 |
| FL_OFC_R | 1.13 | 1.13 | 1.21 | 0.96 | 1.52 | 1.11 | 1.26 | 1.36 | 1.65 | 1.35 |
| FL_Mid_L | 1.12 | 1.11 | 1.05 | 0.97 | 1.59 | 1.02 | 1.56 | 1.17 | 1.33 | 1.45 |
| FL_Mid_R | 1.11 | 1.11 | 1.07 | 0.95 | 1.62 | 1.02 | 1.39 | 1.21 | 1.40 | 1.33 |
| FL_Precen_L | 1.10 | 1.08 | 1.10 | 0.93 | 1.58 | 1.02 | 1.09 | 1.04 | 1.14 | 0.99 |
| FL_Precen_R | 1.13 | 1.10 | 1.11 | 0.92 | 1.59 | 1.03 | 1.11 | 1.05 | 1.18 | 0.93 |
| FL_Inf_L | 1.15 | 1.13 | 1.12 | 0.95 | 1.57 | 1.04 | 1.29 | 1.24 | 1.30 | 1.40 |
| FL_Inf_R | 1.16 | 1.15 | 1.14 | 0.96 | 1.57 | 1.05 | 1.20 | 1.25 | 1.46 | 1.32 |
| FL_Sup_L | 1.11 | 1.09 | 1.09 | 0.93 | 1.55 | 0.98 | 1.31 | 1.11 | 1.30 | 1.16 |
| FL_sup_R | 1.10 | 1.06 | 1.09 | 0.92 | 1.56 | 1.05 | 1.18 | 1.17 | 1.26 | 1.14 |
| PL_L | 1.07 | 1.08 | 1.11 | 0.92 | 1.50 | 0.99 | 1.20 | 1.09 | 1.24 | 1.20 |
| PL_R | 1.10 | 1.06 | 1.13 | 0.90 | 1.51 | 1.02 | 1.14 | 1.11 | 1.31 | 1.06 |
| OL_L | 1.06 | 1.05 | 1.10 | 0.95 | 1.50 | 0.98 | 1.14 | 1.18 | 1.46 | 1.35 |
| OL_R | 1.09 | 1.05 | 1.16 | 0.93 | 1.50 | 1.01 | 1.10 | 1.24 | 1.32 | 1.13 |
| TL_SupLat_L | 1.13 | 1.09 | 1.10 | 0.96 | 1.59 | 1.02 | 1.16 | 1.35 | 1.49 | 1.59 |
| TL_SupLat_R | 1.13 | 1.09 | 1.12 | 0.95 | 1.53 | 1.03 | 1.16 | 1.46 | 1.63 | 1.55 |
| TL_InfLat_L | 1.13 | 1.07 | 1.08 | 0.94 | 1.59 | 1.10 | 1.26 | 1.43 | 1.65 | 1.73 |
| TL_InfLat_R | 1.08 | 1.09 | 1.11 | 0.93 | 1.55 | 1.10 | 1.26 | 1.57 | 1.75 | 1.76 |
| TL_Mesial_L | 1.23 | 1.10 | 1.13 | 0.94 | 1.56 | 1.13 | 1.20 | 1.46 | 1.40 | 1.57 |
| TL_Mesial_R | 1.16 | 1.14 | 1.11 | 0.96 | 1.58 | 1.16 | 1.23 | 1.41 | 1.40 | 1.52 |
| **Mean** | 1.12 | 1.09 | 1.11 | 0.94 | 1.55 | 1.04 | 1.23 | 1.25 | 1.42 | 1.33 |
| **SD** | 0.04 | 0.03 | 0.03 | 0.02 | 0.04 | 0.05 | 0.12 | 0.15 | 0.17 | 0.24 |

**Table 2S:** [^18^F] JNJ-64326067 SUVr averaged between 120 and 180 min in HV and AD subjects. Abbreviations as per Table 1S.

| **Region** | **HV** | | | | | **AD** | | | | |
| --- | --- | --- | --- | --- | --- | --- | --- | --- | --- | --- |
|  | **05** | **09** | **10** | **13** | **15** | **04** | **06** | **08** | **11** | **14** |
| FL_OFC_L | 1.19 | 1.13 | 1.18 | 0.95 | 1.21 | 0.87 | 1.87 | 1.53 | 2.26 | 1.51 |
| FL_OFC_R | 1.19 | 1.13 | 1.17 | 0.95 | 1.22 | 0.88 | 1.57 | 1.63 | 2.34 | 1.43 |
| FL_Mid_L | 1.23 | 1.1 | 1.07 | 0.94 | 1.14 | 0.92 | 2.24 | 1.25 | 1.67 | 1.68 |
| FL_Mid_R | 1.19 | 1.11 | 1.10 | 0.93 | 1.13 | 0.93 | 1.88 | 1.34 | 1.84 | 1.41 |
| FL_Precen_L | 1.21 | 1.08 | 1.13 | 0.94 | 1.16 | 0.93 | 1.34 | 1.24 | 1.41 | 1.21 |
| FL_Precen_R | 1.23 | 1.09 | 1.13 | 0.93 | 1.16 | 0.97 | 1.36 | 1.29 | 1.49 | 1.04 |
| FL_Inf_L | 1.21 | 1.10 | 1.13 | 0.94 | 1.15 | 0.91 | 1.70 | 1.43 | 1.57 | 1.65 |
| FL_Inf_R | 1.22 | 1.11 | 1.18 | 0.94 | 1.16 | 0.95 | 1.53 | 1.51 | 1.89 | 1.41 |
| FL_Sup_L | 1.19 | 1.08 | 1.16 | 0.95 | 1.13 | 0.91 | 1.72 | 1.19 | 1.71 | 1.40 |
| FL_sup_R | 1.15 | 1.06 | 1.20 | 0.93 | 1.11 | 0.95 | 1.44 | 1.32 | 1.71 | 1.23 |
| PL_L | 1.20 | 1.10 | 1.24 | 0.95 | 1.13 | 0.99 | 1.65 | 1.35 | 1.69 | 1.51 |
| PL_R | 1.23 | 1.11 | 1.30 | 0.93 | 1.12 | 1.01 | 1.49 | 1.43 | 1.85 | 1.29 |
| OL_L | 1.14 | 1.07 | 1.30 | 1.03 | 1.19 | 0.99 | 1.58 | 1.47 | 2.17 | 1.67 |
| OL_R | 1.20 | 1.09 | 1.42 | 0.98 | 1.19 | 1.00 | 1.44 | 1.61 | 1.69 | 1.28 |
| TL_SupLat_L | 1.16 | 1.07 | 1.17 | 0.97 | 1.17 | 0.96 | 1.65 | 1.71 | 2.36 | 2.06 |
| TL_SupLat_R | 1.19 | 1.08 | 1.24 | 0.94 | 1.15 | 0.98 | 1.62 | 2.25 | 2.59 | 1.74 |
| TL_InfLat_L | 1.16 | 1.04 | 1.14 | 0.93 | 1.18 | 0.97 | 1.68 | 2.05 | 2.76 | 2.60 |
| TL_InfLat_R | 1.15 | 1.05 | 1.14 | 0.92 | 1.17 | 0.99 | 1.72 | 2.99 | 3.09 | 2.27 |
| TL_Mesial_L | 1.28 | 1.14 | 1.22 | 1.03 | 1.24 | 1.01 | 1.66 | 2.26 | 2.32 | 1.89 |
| TL_Mesial_R | 1.21 | 1.14 | 1.19 | 0.97 | 1.21 | 1.04 | 1.61 | 2.47 | 2.31 | 1.63 |
| **Mean** | **1.20** | **1.09** | **1.19** | **0.95** | **1.16** | **0.95** | **1.64** | **1.62** | **2.02** | **1.59** |
| **SD** | **0.03** | **0.03** | **0.08** | **0.03** | **0.03** | **0.05** | **0.20** | **0.49** | **0.46** | **0.38** |

***SUVr between 120-165 min was used for AD subject 14.**

Table 3S. Comparison of SUVr between AD and HV by region. Timeframe=120-140minutes. a: Cortical, b: subcortical and mesial regions. FL=frontal, OL=occipital, TL=temporal, Cing=cingulate. p-value for 2-sided t-test, assuming unequal variances. Results for right and left were similar, left regions only are provided for clarity.

a.

| **Region** | **N (AD)** | **Mean (AD)** | **StdDev (AD)** | **N (HV)** | **Mean (HV)** | **StdDev (HV)** | **Mean (AD-HV)** | **StdDev (AD-HV)** | **p-value (t-test)** | **Effect Size (Cohen's d)** |
| --- | --- | --- | --- | --- | --- | --- | --- | --- | --- | --- |
| **FL_Inf_L** | 5 | 1.444 | 0.3043 | 5 | 1.112 | 0.1018 | 0.331 | 0.2269 | 0.0701 | 1.461 |
| **FL_Mid_L** | 5 | 1.540 | 0.4678 | 5 | 1.106 | 0.0947 | 0.434 | 0.3375 | 0.1064 | 1.286 |
| **FL_OFC_L** | 5 | 1.598 | 0.4724 | 5 | 1.137 | 0.1110 | 0.461 | 0.3431 | 0.0940 | 1.344 |
| **FL_Precen_L** | 5 | 1.214 | 0.1723 | 5 | 1.115 | 0.0953 | 0.099 | 0.1392 | 0.3031 | 0.710 |
| **FL_Sup_L** | 5 | 1.381 | 0.3310 | 5 | 1.114 | 0.0831 | 0.267 | 0.2413 | 0.1470 | 1.107 |
| **OL_L** | 5 | 1.546 | 0.4025 | 5 | 1.148 | 0.1077 | 0.398 | 0.2946 | 0.0911 | 1.350 |
| **PL_L** | 5 | 1.418 | 0.2748 | 5 | 1.131 | 0.1083 | 0.287 | 0.2088 | 0.0797 | 1.374 |
| **TL_InfLat_L** | 5 | 1.968 | 0.6885 | 5 | 1.091 | 0.1000 | 0.877 | 0.4920 | 0.0457 | 1.782 |
| **TL_Mesial_L** | 5 | 1.760 | 0.4712 | 5 | 1.184 | 0.0984 | 0.576 | 0.3404 | 0.0508 | 1.693 |
| **TL_SupLat_L** | 5 | 1.712 | 0.4964 | 5 | 1.121 | 0.0941 | 0.591 | 0.3573 | 0.0552 | 1.653 |

b.

| **Region** | **N (AD)** | **Mean (AD)** | **StdDev (AD)** | **N (HV)** | **Mean (HV)** | **StdDev (HV)** | **Mean (AD-HV)** | **StdDev (AD-HV)** | **p-value (t-test)** | **Effect Size (Cohen's d)** |
| --- | --- | --- | --- | --- | --- | --- | --- | --- | --- | --- |
| **Hippocampus_L** | 5 | 1.289 | 0.3071 | 5 | 1.019 | 0.1048 | 0.270 | 0.2295 | 0.1224 | 1.179 |
| **Amygdala_L** | 5 | 1.516 | 0.4691 | 5 | 0.969 | 0.1201 | 0.547 | 0.3424 | 0.0579 | 1.598 |
| **Cing_Ant_L** | 5 | 1.411 | 0.4071 | 5 | 1.173 | 0.1165 | 0.238 | 0.2994 | 0.2683 | 0.795 |
| **Cing_Post_L** | 5 | 1.557 | 0.3256 | 5 | 1.215 | 0.1170 | 0.341 | 0.2446 | 0.0784 | 1.395 |
| **Thalamus_L** | 5 | 0.980 | 0.1540 | 5 | 1.009 | 0.1059 | -.029 | 0.1322 | 0.7375 | 0.220 |
| **Putamen_L** | 5 | 1.248 | 0.3039 | 5 | 1.033 | 0.1270 | 0.216 | 0.2329 | 0.1990 | 0.927 |
| **CaudateNucl_L** | 5 | 0.789 | 0.1661 | 5 | 0.885 | 0.1043 | -.096 | 0.1387 | 0.3092 | 0.696 |
| **Pallidum_L** | 5 | 1.163 | 0.2702 | 5 | 1.048 | 0.1601 | 0.115 | 0.2221 | 0.4432 | 0.516 |

Table 4S. Comparison of DVR between AD and HV by region. a: Cortical, b: subcortical and mesial regions. Abbreviations as in Table 3S. Results for right and left were similar, left regions only are provided for clarity.

a.

| **Region** | **N (AD)** | **Mean (AD)** | **StdDev (AD)** | **N (HV)** | **Mean (HV)** | **StdDev (HV)** | **Mean (AD-HV)** | **StdDev (AD-HV)** | **p-value (t-test)** | **Effect Size (Cohen's D)** |
| --- | --- | --- | --- | --- | --- | --- | --- | --- | --- | --- |
| **FL_Inf_L** | 5 | 1.284 | 0.1711 | 5 | 1.084 | 0.0731 | 0.200 | 0.1316 | 0.0578 | 1.518 |
| **FL_Mid_L** | 5 | 1.331 | 0.2416 | 5 | 1.082 | 0.0604 | 0.248 | 0.1761 | 0.0821 | 1.411 |
| **FL_OFC_L** | 5 | 1.368 | 0.2712 | 5 | 1.077 | 0.0831 | 0.291 | 0.2006 | 0.0730 | 1.452 |
| **FL_Precen_L** | 5 | 1.101 | 0.0871 | 5 | 1.069 | 0.0671 | 0.032 | 0.0778 | 0.5309 | 0.415 |
| **FL_Sup_L** | 5 | 1.201 | 0.1707 | 5 | 1.066 | 0.0656 | 0.135 | 0.1293 | 0.1576 | 1.045 |
| **OL_L** | 5 | 1.261 | 0.2582 | 5 | 1.055 | 0.0597 | 0.205 | 0.1874 | 0.1512 | 1.096 |
| **PL_L** | 5 | 1.194 | 0.1354 | 5 | 1.061 | 0.0700 | 0.134 | 0.1078 | 0.0978 | 1.239 |
| **TL_InfLat_L** | 5 | 1.491 | 0.4377 | 5 | 1.040 | 0.0752 | 0.452 | 0.3141 | 0.0815 | 1.439 |
| **TL_Mesial_L** | 5 | 1.356 | 0.2512 | 5 | 1.046 | 0.0786 | 0.311 | 0.1862 | 0.0482 | 1.669 |
| **TL_SupLat_L** | 5 | 1.355 | 0.3093 | 5 | 1.060 | 0.0644 | 0.294 | 0.2234 | 0.1003 | 1.316 |

b.

| **Region** | **N (AD)** | **Mean (AD)** | **StdDev (AD)** | **N (HV)** | **Mean (HV)** | **StdDev (HV)** | **Mean (AD-HV)** | **StdDev (AD-HV)** | **p-value (t-test)** | **Effect Size (Cohen's D)** |
| --- | --- | --- | --- | --- | --- | --- | --- | --- | --- | --- |
| **Hippocampus_L** | 5 | 1.139 | 0.1381 | 5 | 1.011 | 0.0670 | 0.129 | 0.1086 | 0.1113 | 1.187 |
| **Amygdala_L** | 5 | 1.265 | 0.2206 | 5 | 0.992 | 0.0694 | 0.273 | 0.1636 | 0.0478 | 1.672 |
| **Cing_Ant_L** | 5 | 1.245 | 0.2287 | 5 | 1.124 | 0.0781 | 0.120 | 0.1709 | 0.3172 | 0.704 |
| **Cing_Post_L** | 5 | 1.338 | 0.1540 | 5 | 1.170 | 0.0769 | 0.168 | 0.1217 | 0.0731 | 1.378 |
| **Thalamus_L** | 5 | 1.096 | 0.1114 | 5 | 1.176 | 0.0782 | -.079 | 0.0962 | 0.2319 | 0.826 |
| **Putamen_L** | 5 | 1.305 | 0.2185 | 5 | 1.204 | 0.0873 | 0.100 | 0.1664 | 0.3821 | 0.603 |
| **CaudateNucl_L** | 5 | 0.791 | 0.1829 | 5 | 0.903 | 0.1344 | -.112 | 0.1605 | 0.3030 | 0.701 |
| **Pallidum_L** | 5 | 1.293 | 0.1915 | 5 | 1.241 | 0.1057 | 0.052 | 0.1547 | 0.6142 | 0.335 |


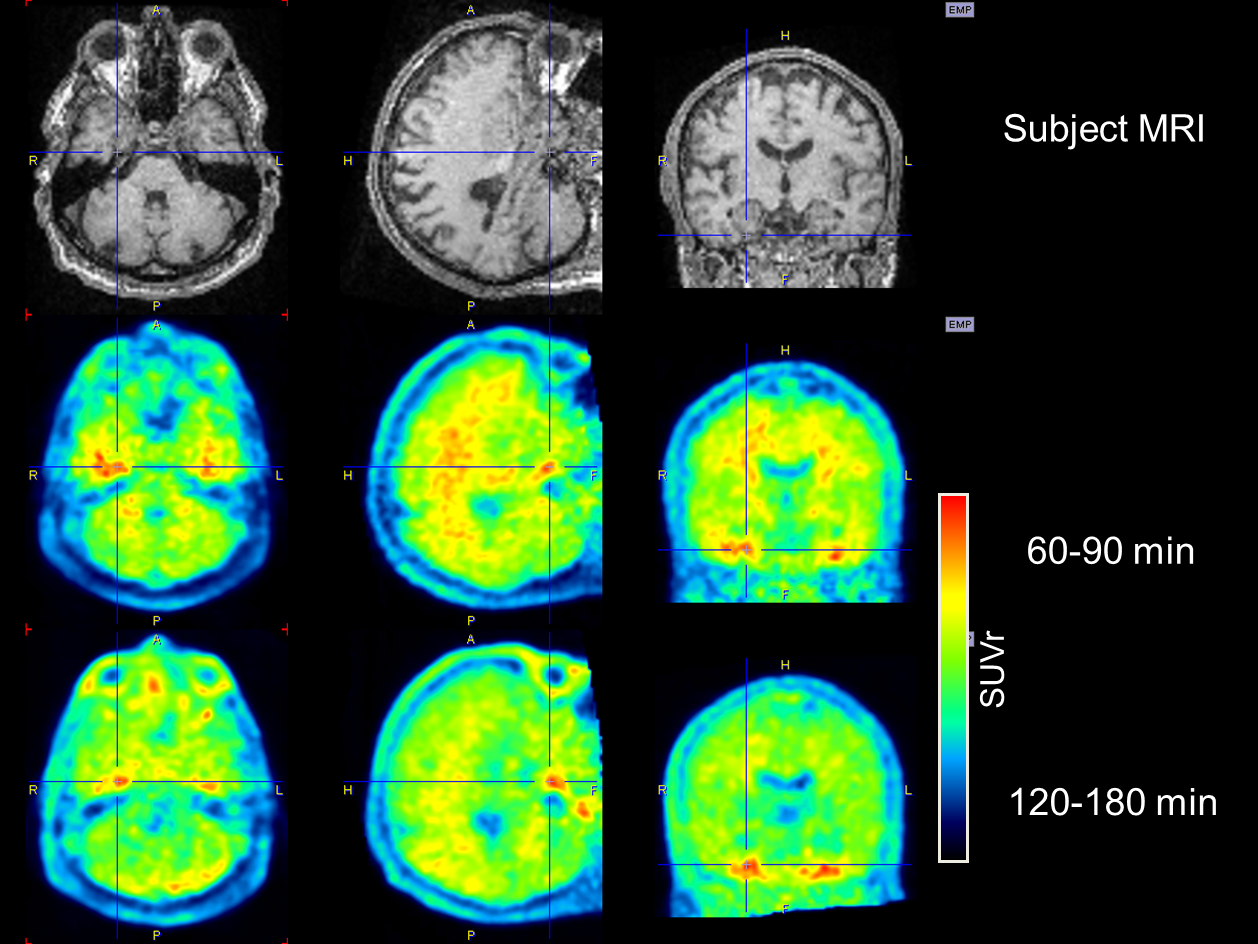

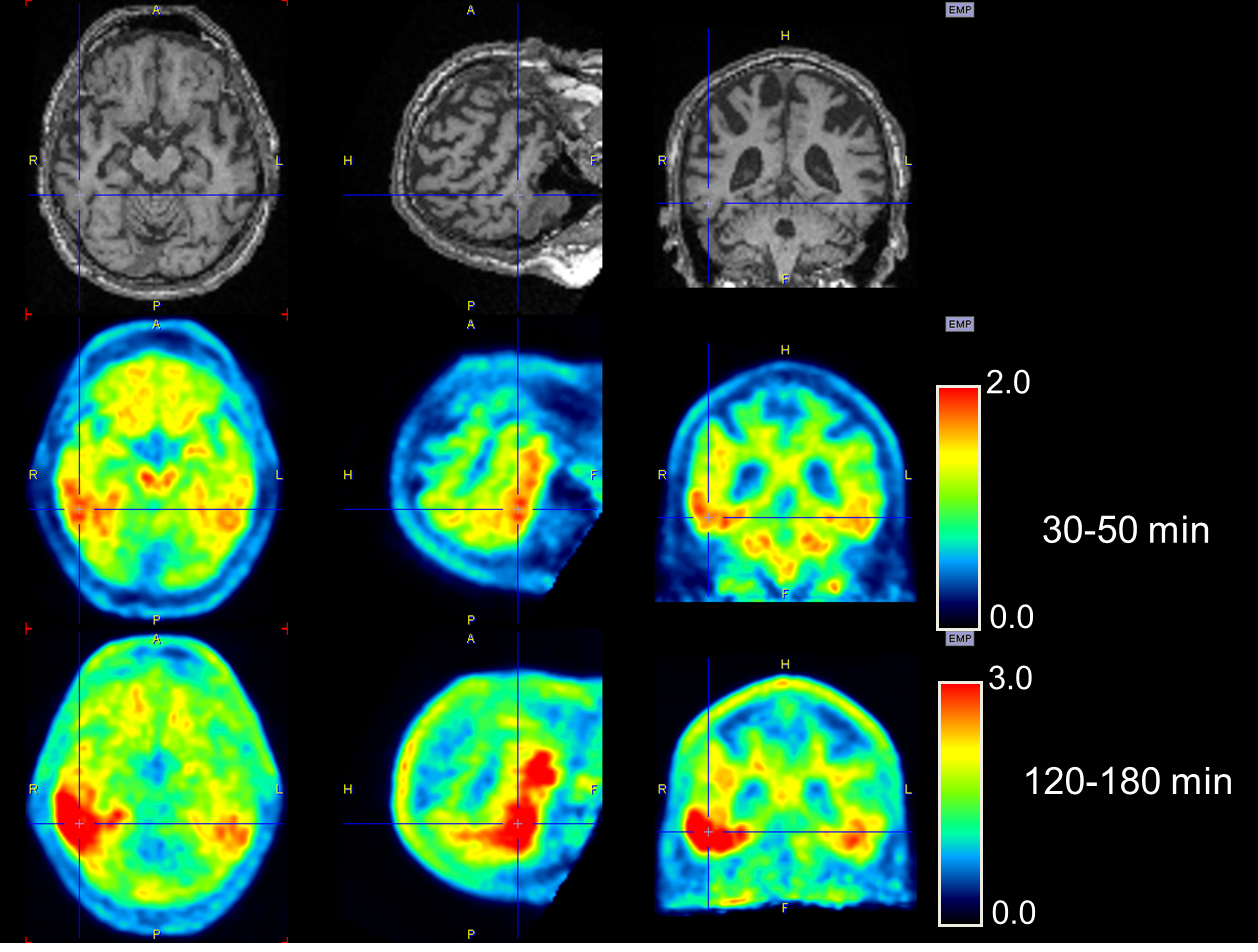


AD Subject 4

1.8

0.0

AD Subject 8


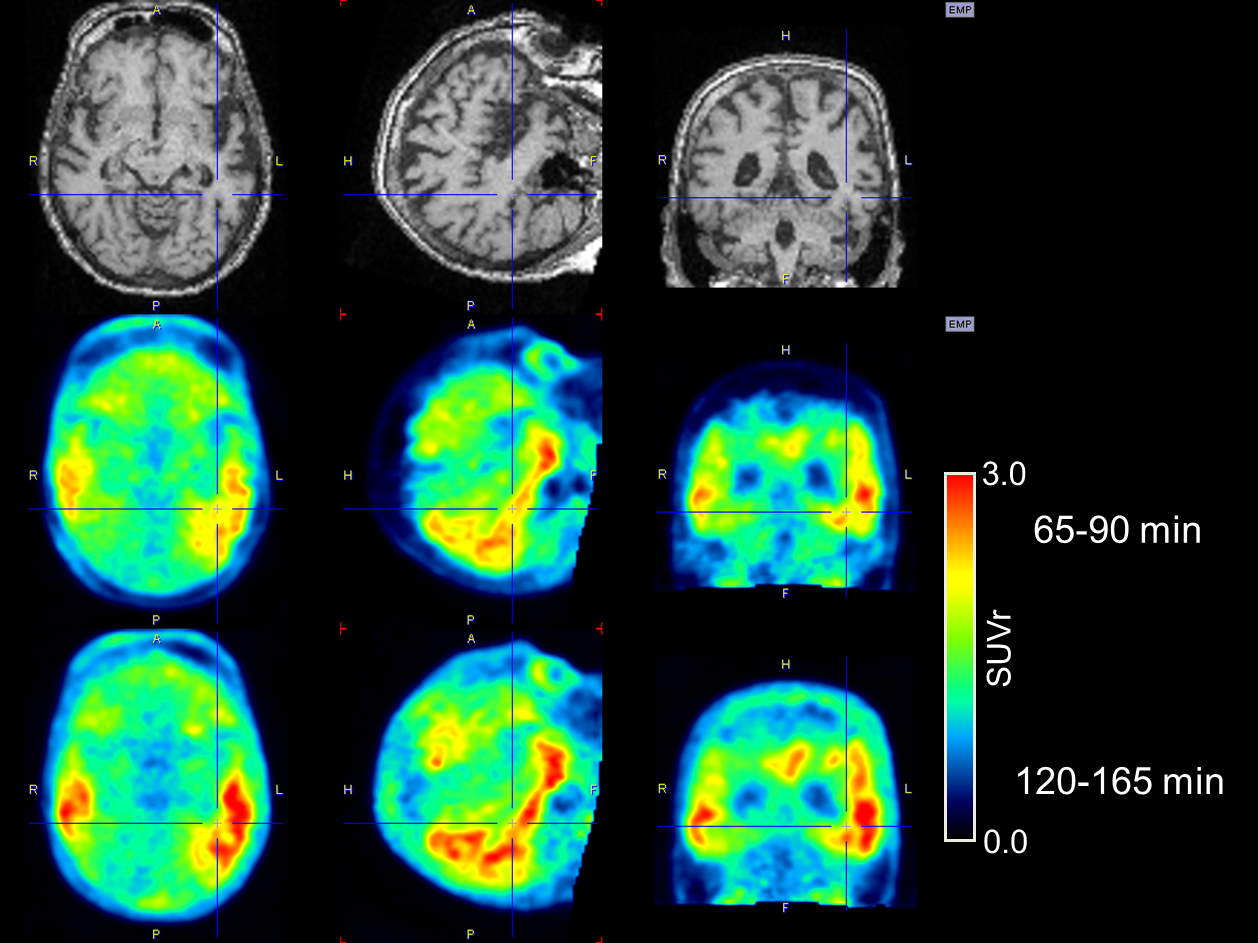

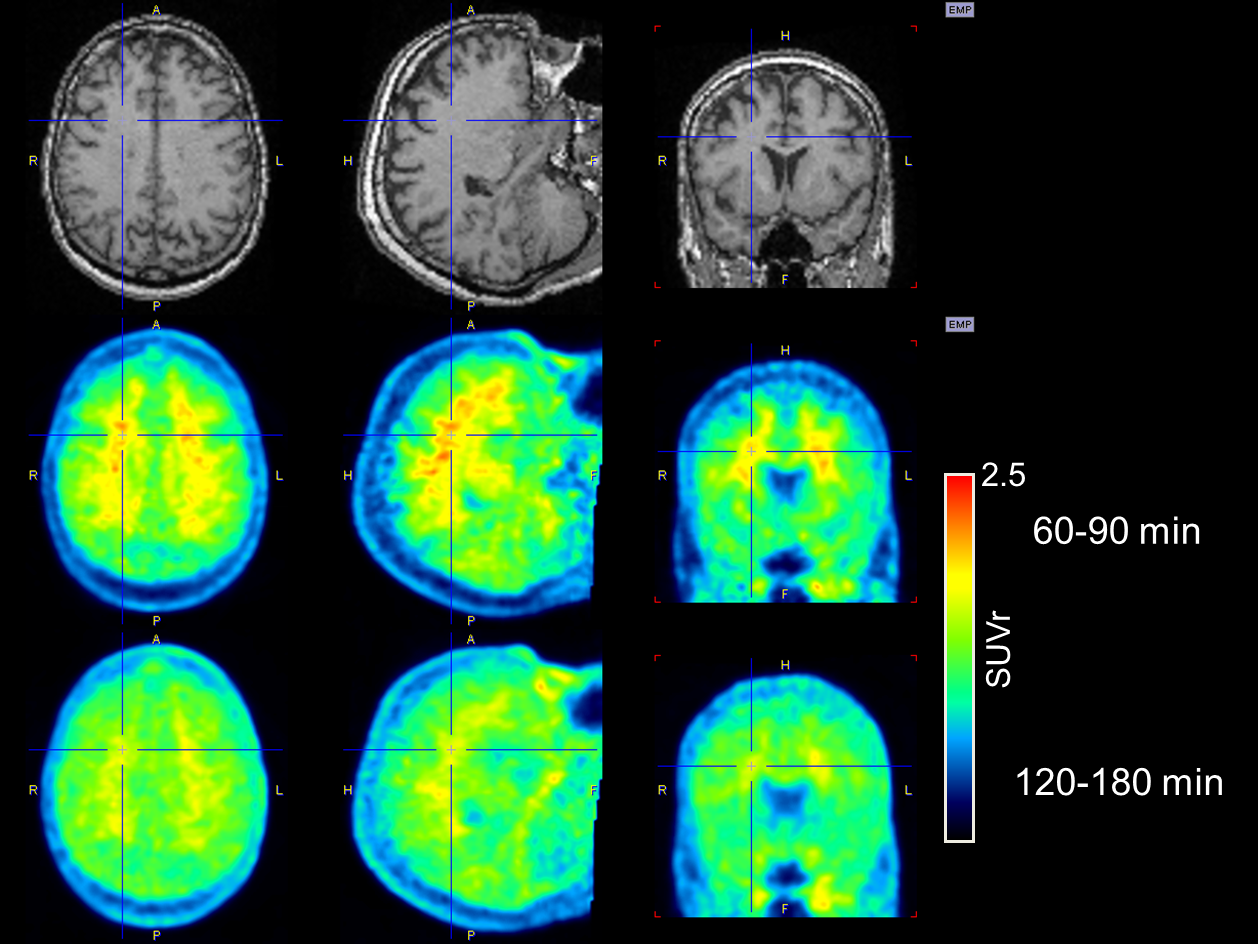


HV Subject 5

AD Subject 14


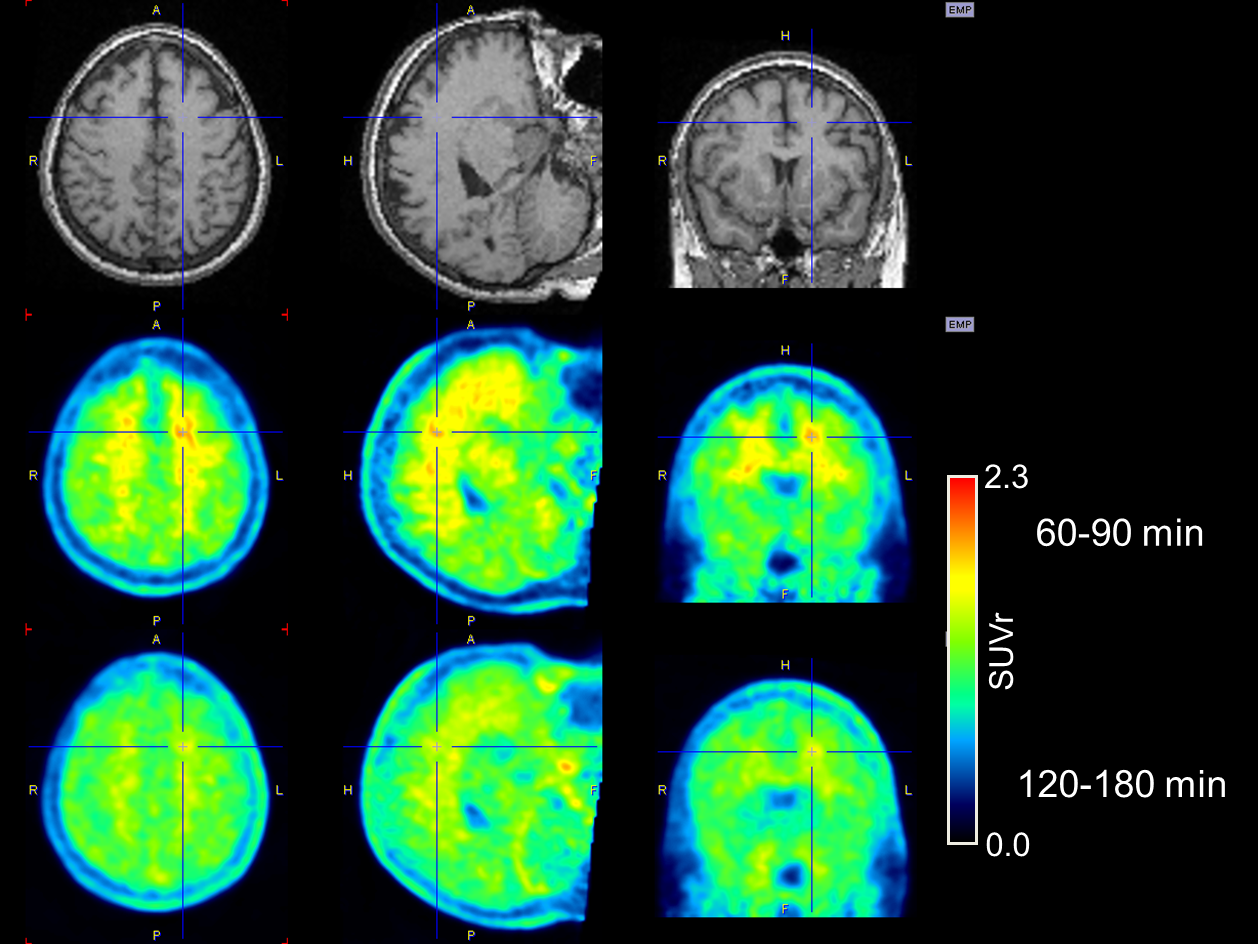

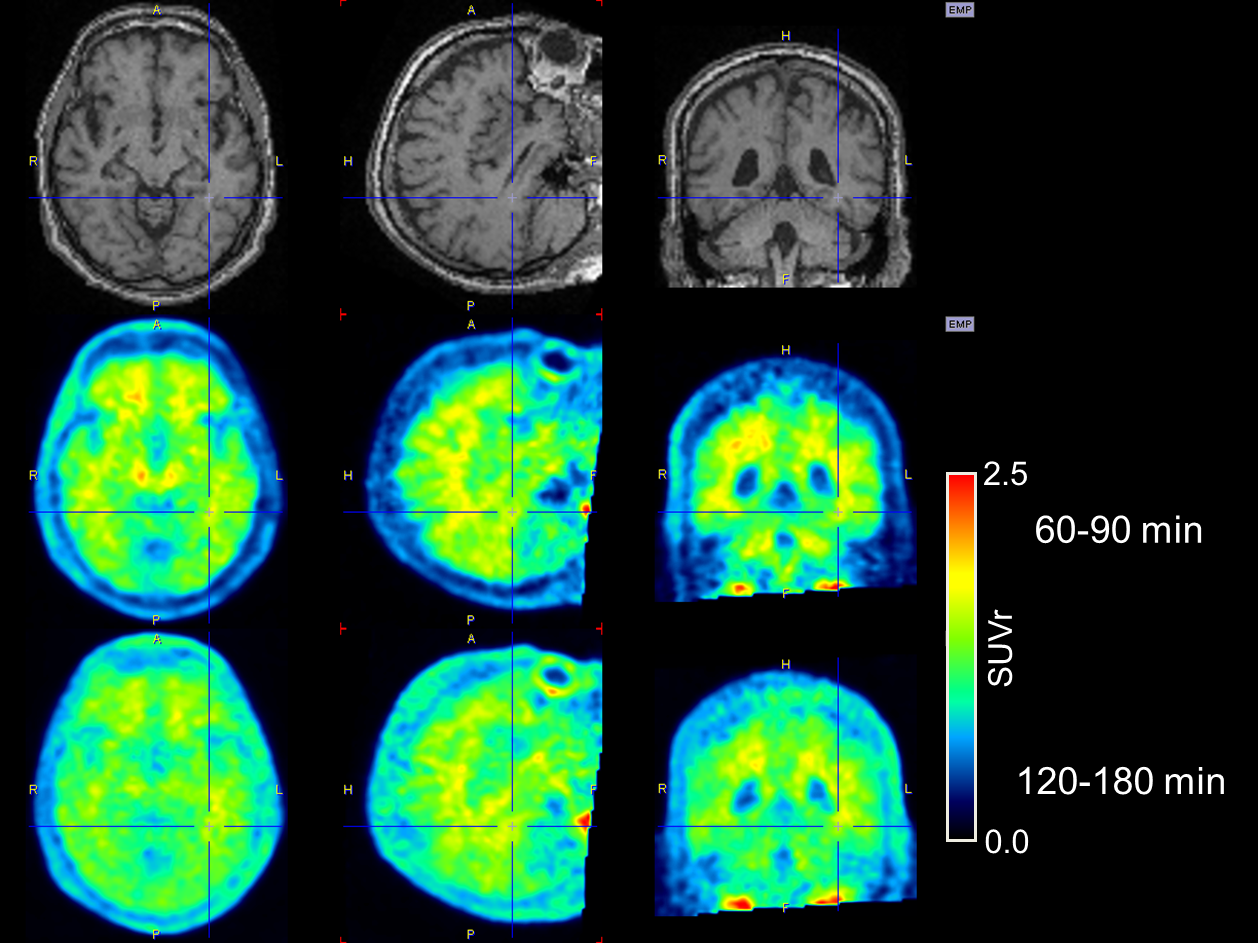


HV Subject 15

HV Subject 9

Figure 2S. Individual MRI (top rows), 60-90 min (middle rows) and 120-180 min (bottom rows) [^18^F] JNJ-64326067 PET SUVr images for AD subject 4 and HV subjects 5,9, and 15. The summed 30-35 min frames for AD subject 8 and summed 65-90 min and 120-165 frames for AD subject 14 are presented due to exclusion of frames with significant motion. AD subject 4 was read as visually negative.

Figure 3S. Representative Logan plots for AD subject 11 with a fit starting time (t*) at 25 min, using ventral cerebellar cortex as a reference region

Figure 4S. A small VOI was placed on the skull of subject HV 05 (appears dark on T-1 weighted MR image) and the TAC was generated below. Activity is low in the skull and there was no evidence of defluorination of [^18^F]JNJ-64326067 observed based on the TAC kinetics. Uptake observed outside of the brain appears to be in the scalp.


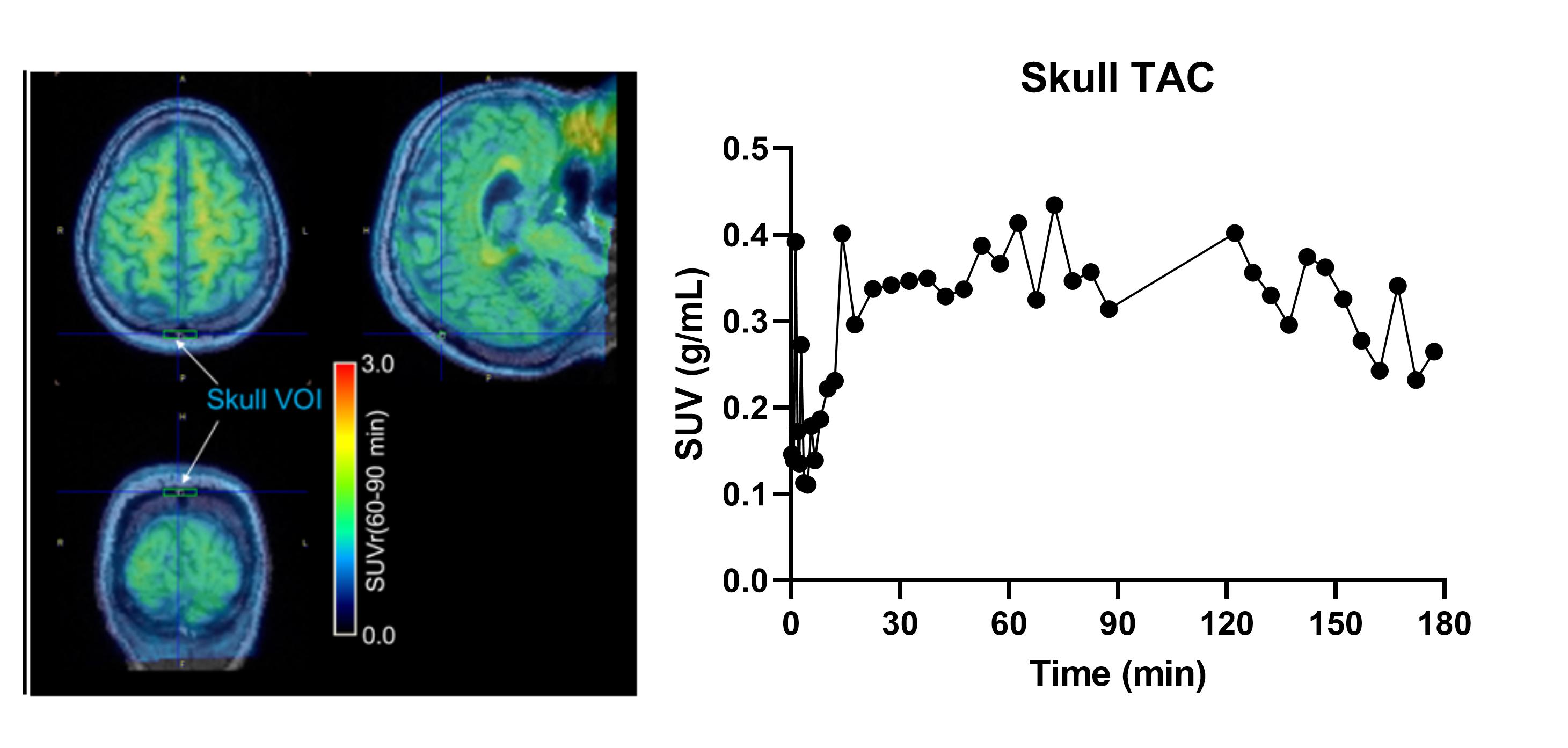


Figure 5S. Representative [^18^F] JNJ-64326067 SUV and SUVr time-activity curves for HV subjects. Abbreviations: medial frontal cortex (FL_Mid_L), inferior lateral temporal cortex (TL_InfLat_L), posterior cingulate (Cing_Post_L). The left occipital region was manually drawn around an area of high visual signal in HV 10, who had a shotgun injury to the back of the head due to a hunting accident during adolescence.

Figure 6S. Representative [^18^F] JNJ-64326067 SUV and SUVr time-activity curves for AD subjects. Abbreviations: medial frontal cortex (FL_Mid_L), inferior lateral temporal cortex (TL_InfLat_L), posterior cingulate (Cing_Post_L). AD subject 04 was visually read as negative. Frames in subjects AD 08 and AD 14 were deleted due to excessive between frame movement. AD 08 had a MMSE of 12 and CDR 2, AD 14 had a MMSE of 23 and CDR 1 (Table 1).

# References

1. Human alimentary tract model for radiological protection. ICRP Publication 100. A report of The International Commission on Radiological Protection. Ann.ICRP 2006;36(1-2):25-327, iii.
2. Ziessman HA, Jones DA, Muenz LR, Agarval AK. Cholecystokinin cholescintigraphy: methodology and normal values using a lactose-free fatty-meal food supplement. J Nucl Med 2003 Aug;44(8):1263-6.
3. Stabin MG, Sparks RB, Crowe E. OLINDA/EXM: the second-generation personal computer software for internal dose assessment in nuclear medicine. J.Nucl.Med. 2005 Jun;46(6):1023-1027.
4. Basic anatomical and physiological data for use in radiological protection: reference values. A report of age- and gender-related differences in the anatomical and physiological characteristics of reference individuals. ICRP Publication 89. Ann.ICRP 2002;32(3-4):5-265.
5. The 2007 Recommendations of the International Commission on Radiological Protection. ICRP publication 103. Ann.ICRP 2007;37(2-4):1-332.
